# Disruption of *bbe02* by Insertion of a Luciferase Gene Increases Transformation Efficiency of *Borrelia burgdorferi* and Allows Live Imaging in Lyme Disease Susceptible C3H Mice

**DOI:** 10.1371/journal.pone.0129532

**Published:** 2015-06-12

**Authors:** Kamfai Chan, Laura Alter, Stephen W. Barthold, Nikhat Parveen

**Affiliations:** 1 Department of Microbiology, Biochemistry and Molecular Genetics, Rutgers New Jersey Medical School, Newark, NJ, 07103, United States of America; 2 Department of Pathology, Microbiology & Immunology, University of California School of Veterinary Medicine, Davis, CA, 95616, United States of America; University of Toledo School of Medicine, UNITED STATES

## Abstract

Lyme disease is the most prevalent tick-borne disease in North America and Europe. The causative agent, *Borrelia burgdorferi* persists in the white-footed mouse. Infection with *B*. *burgdorferi* can cause acute to persistent multisystemic Lyme disease in humans. Some disease manifestations are also exhibited in the mouse model of Lyme disease. Genetic manipulation of *B*. *burgdorferi* remains difficult. First, *B*. *burgdorferi* contains a large number of endogenous plasmids with unique sequences encoding unknown functions. The presence of these plasmids needs to be confirmed after each genetic manipulation. Second, the restriction modification defense systems, including that encoded by *bbe02* gene lead to low transformation efficiency in *B*. *burgdorferi*. Therefore, studying the molecular basis of Lyme pathogenesis is a challenge. Furthermore, investigation of the role of a specific *B*. *burgdorferi* protein throughout infection requires a large number of mice, making it labor intensive and expensive. To overcome the problems associated with low transformation efficiency and to reduce the number of mice needed for experiments, we disrupted the *bbe02* gene of a highly infectious and pathogenic *B*. *burgdorferi* strain, N40 D10/E9 through insertion of a firefly luciferase gene. The *bbe02* mutant shows higher transformation efficiency and maintains luciferase activity throughout infection as detected by live imaging of mice. Infectivity and pathogenesis of this mutant were comparable to the wild-type N40 strain. This mutant will serve as an ideal parental strain to examine the roles of various *B*. *burgdorferi* proteins in Lyme pathogenesis in the mouse model in the future.

## Introduction

Lyme disease is a multisystemic disease caused by the spirochete, *Borrelia burgdorferi*, which is transmitted to mammalian hosts and birds by ticks primarily belonging to *Ixodes* species. Lyme disease is prevalent in two continents, North America and Europe [1–6]. Infection with this spirochete results in acute to chronic disease manifestations [3]. The white-footed mouse, *Peromyscus leucopus*, is the natural reservoir host of *B*. *burgdorferi*. Different strains of inbred mice (*Mus musculus*) demonstrate different severity of Lyme disease [7]. C3H mice exhibit carditis and arthritic manifestations similar to humans [8]. Therefore, the C3H mouse model has been used extensively to study the molecular basis of tissue colonization by *B*. *burgdorferi* and the resulting disease. Assessing the role of a specific *B*. *burgdorferi* protein in pathogenesis and tissue tropism of spirochetes in mice from acute to persistent stages of infection requires the use of a large number of animals and thus, remains expensive and labor intensive. Therefore, there is an urgent need for techniques that facilitate reduction in the number of mice needed to investigate the role of a particular *B*. *burgdorferi* protein throughout infection.

Bioluminescence is emerging as an important tool for live imaging of pathogens both *in vitro* and *in vivo* [9]. Two main groups of enzymes that are used extensively are: (a) bacterial luciferases produced by *Vibrio fisheri*, *Vibrio harveyi*, and *Photorhabdus luminescens* (*lux* system) that respond to FMNH2, long-chain aldehyde and molecular oxygen, and (b) insect luciferase system from firefly *Photinus pyralis* (*luc* enzyme)*;* or beetle *Pyrophorus plagiophthalamus* that produce light using luciferin in the presence of oxygen and ATP [10–14]. Real time imaging has been employed to study the spatiotemporal progression of bacterial infections in living hosts, and for determining bacterial load and metabolic activity in different tissues during infection [15]. In one of the earliest bioluminescence *in vivo* imaging studies, Contag and coworkers utilized luciferase from *P*. *luminescens* to monitor dissemination of *Salmonella typhimurium* to different tissues and its colonization of specific organs. They could even distinguish progressive versus abortive infection in BALB/c mice [16]. Although *P*. *luminescens* luciferase system is often found to be more sensitive [11, 14], the genes in the *lux* operon encode proteins that are involved in fatty aldehyde substrate synthesis making the system significantly energy expensive [17, 18]. Therefore, this system may not be very efficient for highly auxotrophic, slow growing bacteria, such as Lyme spirochetes. Recently, *in vivo* imaging has been used to monitor the presence, dissemination and tissue colonization of *B*. *burgdorferi* in live hosts, [19–21]. Expansion of these strategies to different *B*. *burgdorferi* strains could significantly improve mouse infection studies allowing assessment of Lyme pathogenesis in real time and hence, revolutionize the field.

Bacteria possess a variety of defense systems, such as restriction modification systems and toxin-antitoxin systems [22]. Restriction modification systems are integral components of bacterial defense schemes and consist of genes encoding restriction enzymes that are often associated with the modifying enzyme with methylase activity which modifies specific sequence of self DNA [23]. By identifying “self” DNA modification at a particular nucleotide, bacterial restriction system can distinguish incoming foreign DNA in the form of plasmid or viral DNA that is either unmethylated or is methylated at different site [24]. The restriction modification complexes then attack the foreign DNA and degrade it. Although genetic manipulation of *B*. *burgdorferi* is now possible, molecular characterization of Lyme spirochetes suffers from low transformation efficiency particularly when the plasmids amplified and purified from *E*. *coli* strains are used to transform infectious *B*. *burgdorferi* strains [25, 26]. *B*. *burgdorferi* possesses a relatively small (1.56Mb) but rather complex genome. Approximately a third of the genome of the sequenced B31 strain consists of 21 linear and circular plasmids. While plasmid content varies among different *B*. *burgdorferi* strains, a comprehensive review of genomes of different strains showed that the repertoire of genes is relatively consistent and thus, maintains tick-mammalian cycle of *B*. *burgdorferi* in nature [27–30]. Previous studies have attributed lower transformation efficiency of *B*. *burgdorferi* to the presence of Type II restriction modification systems encoded by *bbe02* and *bbq67* genes, which are located on the linear endogenous plasmids lp25 and lp56, respectively of the sequenced B31 strain [31–35]. Indeed, the absence of both *bbe02* and *bbq67* has been shown to increase transformation efficiency of infectious *B*. *burgdorferi*. Moreover their loss did not affect spirochetal infectivity or pathogenesis in the mouse model either by injection using needle and syringe, or natural, tick-mediated infection [32, 33].

The lp25 plasmid is found more consistently in different *B*. *burgdorferi* sensu stricto infectious strains while lp56 and hence, *bbq67* is absent in many strains, including the infectious, sequenced N40 strain [27, 34]. Since lp56 is obtained through duplication and assembly of a cp32 plasmid with a linear plasmid [36, 37], the majority of genes located on this plasmid may display redundant functions and hence, are not essential. Interestingly, *bbe22* located on lp25 plasmid encodes nicotinamidase (PncA), an enzyme essential for infectivity of *B*. *burgdorferi* [38]. Thus, lp25 is always present when the live spirochetes are recovered from mice [39]. Using this characteristic, several laboratories have used B31 derivative strains lacking either lp25 or lp56 plasmids for genetic manipulations [21, 38, 40–44]. The specific mutants generated in the strain lacking lp25 are then complemented with the gene(s) of interest cloned in the shuttle vector containing the essential *bbe22* gene such that the plasmid is maintained throughout infection in mice. In a recent *in vivo* imaging study, this strategy was utilized both to express codon-optimized *P*. *pyralis* luciferase in *B*. *burgdorferi* [45], and to study the role of DbpA-DbpB and *BBK32* adhesins in colonization of tissues of infected BALB/c mice [21]. As an alternative, we wanted to develop an infectious *B*. *burgdorferi* strain in which luciferase expression does not require the loss of an endogenous plasmid. Since B31 and N40 are used extensively to study Lyme pathogenesis [46], we decided to use these two strains in this study. We compared two approaches to determine if the firefly luciferase gene located on the *B*. *burgdorferi* shuttle vector [45] in the infectious *B*. *burgdorferi* strain B31, or stable expression of this gene in the highly infectious strain N40 is better for live imaging of the infected mice.

Although the B31 strains lacking lp25 or lp56 have been used for generating specific mutants [21, 38, 40–44], the loss of an endogenous plasmid could affect an as yet unidentified spirochetal function, such as survival of *B*. *burgdorferi* in nature through tick-host transmission cycle [33]. The major aim of this study was to generate a highly infectious, bioluminescent *B*. *burgdorferi* strain that can be examined by real-time imaging, retains its endogenous plasmids and shows higher transformation efficiency. We expected that stable expression of codon-optimized firefly luciferase [45] would allow live imaging of acute to persistent infection using an *in vivo* imaging system (IVIS). Therefore, such a strain can be used as a parental strain to generate mutants defective in specific genes and investigate their role in Lyme pathogenesis by real time imaging, in the future. In fact, *bbe02* disruption using *flgBp-lacI* to stably express LacI was first used to increase transformation efficiency of infectious *B*. *burgdorferi* strain [47]. Using a similar strategy, we generated a derivative strain of *B*. *burgdorferi* N40D10/E9 in which the luciferase gene was inserted, in tandem with the antibiotic resistance marker, into *bbe02* present on the lp25 plasmid. Due to the essential role played by PncA in the survival of *B*. *burgdorferi* during infection [38], we hypothesized that the luciferase gene would not be lost during infection. Moreover, disruption of *bbe02* gene, a putative restriction-modification gene, was also expected to significantly increase the transformation efficiency of the bioluminescent *B*. *burgdorferi* strain [32]. Indeed, our bioluminescent N40 strain retains the lp25 plasmid and the presence of live spirochetes can be examined throughout infection in the mice. The strain also exhibits higher transformation efficiency.

## Materials and Methods

### Bacterial strains and plasmid

**Fig 1:**
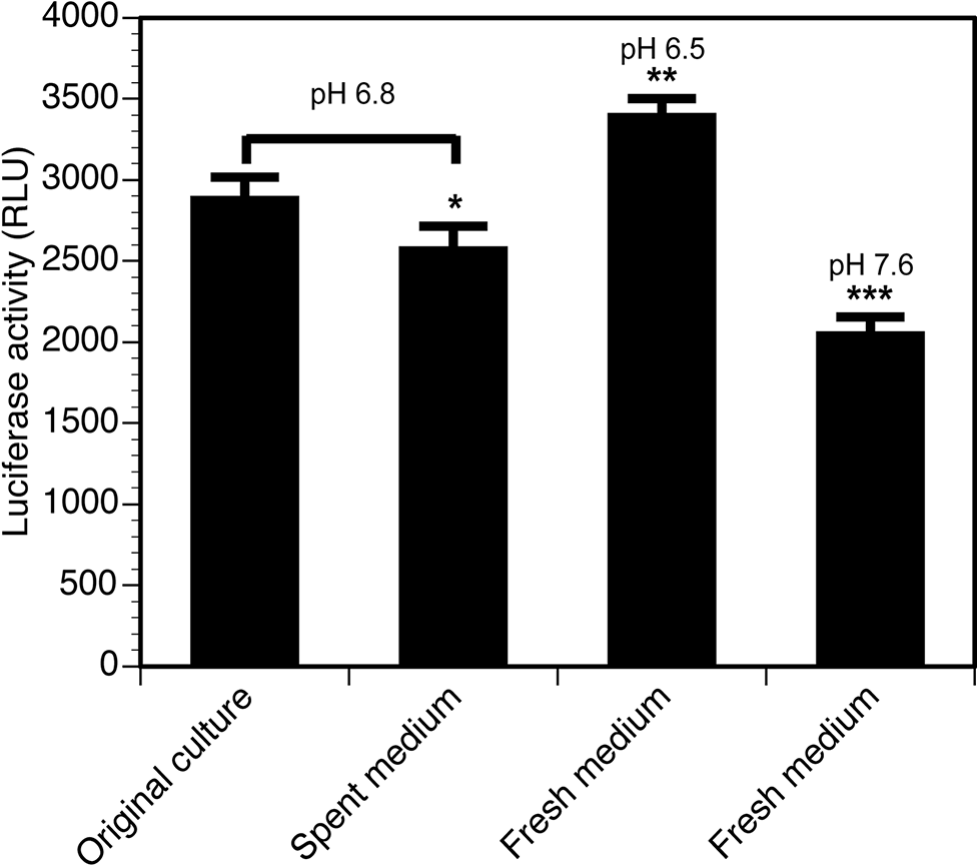
Codon-optimized firefly luciferase activity in *B*. *burgdorferi* is affected by both pH and availability of energy source. Fresh *B*. *burgdorferi* medium adjusted to pH 6.5 appears most optimal for D-luciferin hydrolysis and light emission by luciferase enzyme. Aliquots from the late log-stationary phase culture replicates were prepared and after centrifugation, pellets were resuspended in fresh medium, fresh medium adjusted to pH6.5, or spent medium at pH6.8. One aliquot each from culture replicate (total four) was included as control. Readily available energy source remaining in the culture medium in which *B*. *burgdorferi* has been grown to early stationary phase growth (pH 6.8) was sufficient to emit light significantly higher than fresh medium (pH 7.6) containing the same number of spirochetes. Four replicates were used for each treatment. Statistically significant differences were determined by t-Test for two samples with unequal variances and are depicted by asterisks in the figure (* P<0.05, ** P<0.001, and *** P<0.0001).

Low passage, infectious *B*. *burgdorferi* strains were used in this study. Strain N40 clone D10/E9 was originally cloned and provided by John Leong at Tufts University Medical School, Boston. Strain B31 clones 5A4 and 5A18 NP1 were obtained from the laboratory of Steven Norris at University of Texas, Houston. *B*. *burgdorferi* was cultured at 33°C in Barbour-Stoenner-Kelly-II (BSK-II) medium supplemented with 6% rabbit serum, i.e., BSKII+RS [48]. Endogenous plasmid profiles of these strains and their transformants (data not shown) were determined by PCR as described previously [49, 50]. To determine if a readily available energy source or pH of the medium affect the luciferase activity, we determined light emission by late logarithmic to early stationary phase culture replicates of *B*. *burgdorferi* without centrifugation in four replicate cultures. Four replicates of each treatment were used to facilitate statistical analysis. Three aliquots from each culture replicate were also centrifuged and resuspended in the spent medium without pH adjustment, and fresh medium with normal pH 7.6, or in fresh medium with the pH adjusted to 6.5 (Fig 1). Spirochetes were adjusted to the same number in different treatments.

**Fig 2:**
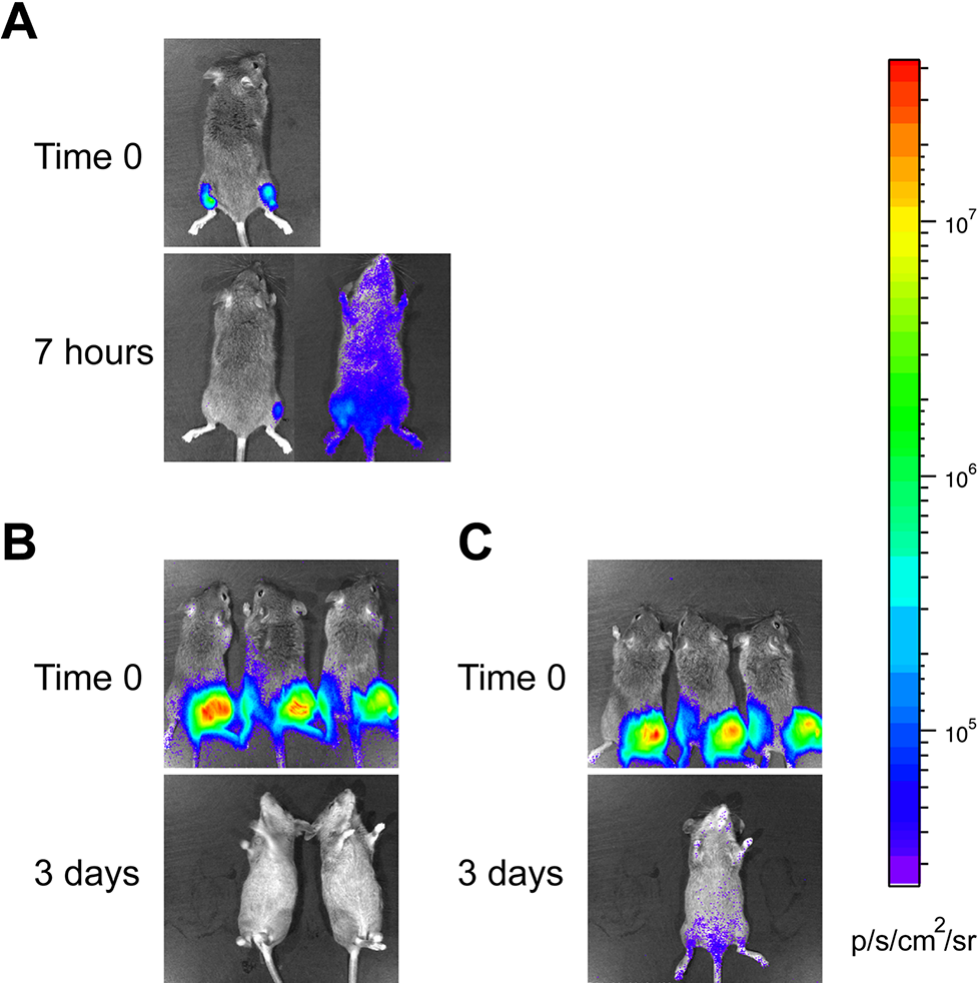
Shuttle vector containing *Bbluc* is unstable during infection of mice in the absence of the antibiotic selection. **(A)** C3H mice were inoculated subcutaneously with approximately 10^6^ *B*. *burgdorferi* B31 strain harboring the shuttle vector pJSB175. Ten minutes after injection of the spirochetes along with 1.5 mg of D-luciferin, bioluminescence was detected using IVIS. Further imaging was done 7 hours later. **(B and C)** In the second experiment, ten minutes after injection of 10^6^ *B*. *burgdorferi* B31 possessing pJSB175 with 1.5 mg of D-luciferin in six C3H mice subcutaneously, bioluminescence was detected using IVIS in groups of three mice each. **(B)** One group was provided with drinking water without antibiotic, and **(C)** drinking water for the second group of mice contained 5 mg/ml streptomycin. Bioluminescent images were taken 1 day (not shown) and 3 days after infection. Loss of light emission likely due to the loss of plasmid in the absence of selection was observed (B). The same minimum and maximum luminescence range were used in all images as indicated by color spectrum scale.

The *B*. *burgdorferi*-codon optimized firefly luciferase gene (*Bbluc*) under the control of a *flaB* promoter carried on a shuttle vector pJSB175 exhibits bioluminescence in *B*. *burgdorferi* [45]. We used this strategy to express luciferase in B31 strain (Fig 2). DNA fragment containing this gene and Str^R^ cassette [51] was used to generate mutation of the *bbe02* gene located on the lp25 plasmid in the N40 strain.

### Construction of plasmid clone to generate *bbe02* mutant strains

**Fig 3:**
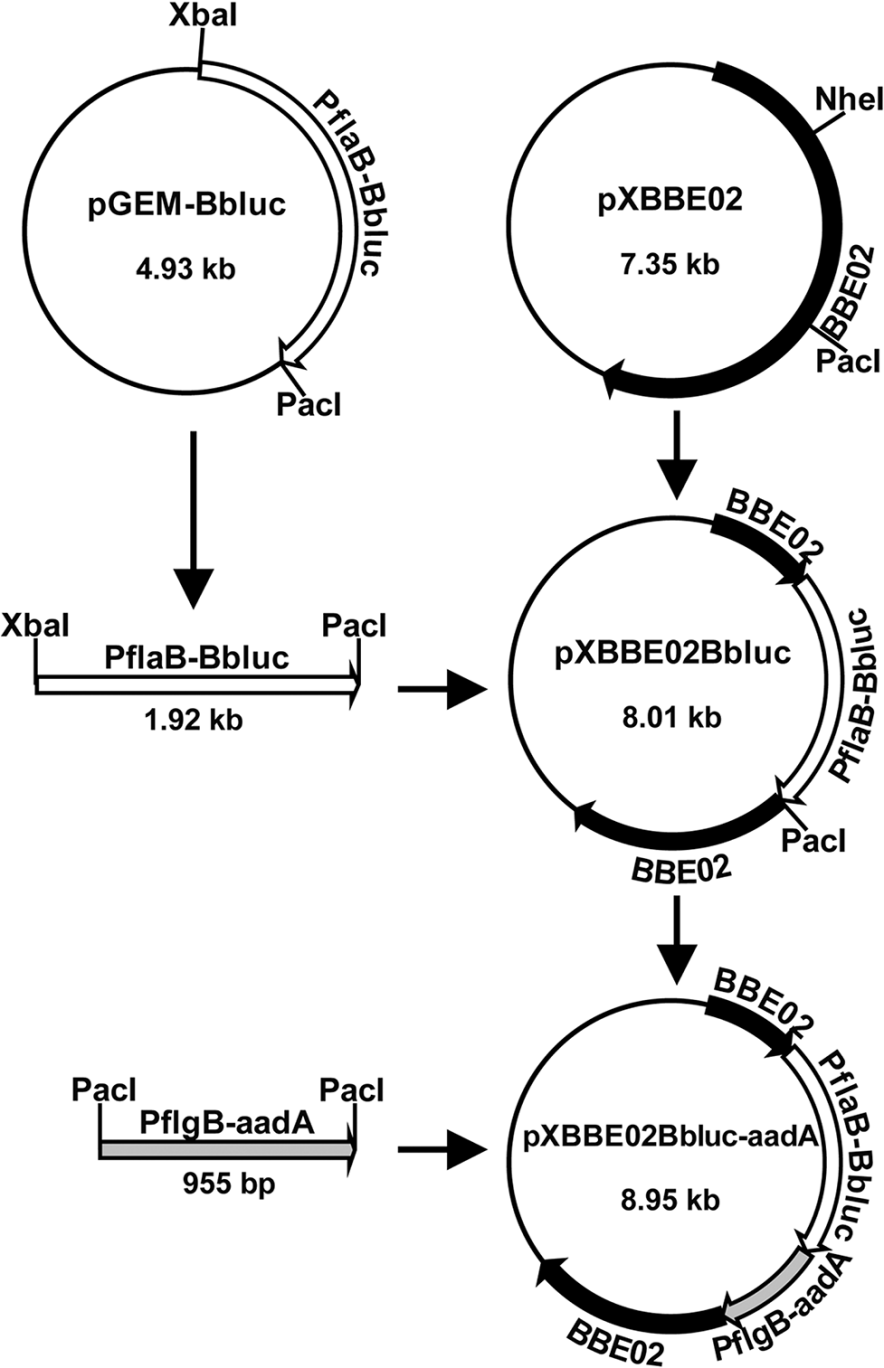
Generation of suicide plasmid for mutagenesis in which *B*. *burgdorferi*-codon optimized firefly luciferase gene and streptomycin resistance cassette in tandem disrupt *bbe02* gene. The *bbe02* gene was amplified from the B31 genome using primers BBE02Nde-long and BBE02Xho-long, and amplicon was cloned into pCR-XL-TOPO vector to form pX*bbe02*. The plasmid pX*bbe02* digested with NheI and PacI restriction enzymes liberated the internal fragment of the *bbe02* gene. *B*. *burgdorferi*-codon optimized firefly luciferase gene under the *flaB* promoter (P*flaB*-*Bbluc*) amplified from plasmid pJSB175 was then cloned using XbaI and PacI restriction enzymes, in the compatible NheI and PacI overhangs of the pX*bbe02* to form pX*bbe02Bbluc*. The streptomycin resistance cassette was then cloned into the PacI site at the 3’ end of the *Bbluc* gene, to form pX*bbe02Bbluc-aadA*. This plasmid was then used to transform infectious *B*. *burgdorferi* to generate bioluminescent N40 strain with disrupted *bbe02* gene.

The cloning strategy to generate *bbe02* mutant is depicted in Fig 3. Briefly, the *bbe02* gene was amplified using the primers BBE02Nde-long and BBE02Xho-long (Table 1). A ~3.8 kb PCR product so obtained was then cloned into the pCR-XL-TOPO vector (Life Technologies, NY) to obtain the pX*bbe02* plasmid and sequenced to confirm the inserted gene to be *bbe02*. Restriction digestion of this plasmid with NheI and PacI removed an internal 1248 bp segment of the *bbe02* gene. The codon-optimized firefly luciferase gene under *flaB* promoter (P*flaB*-*Bbluc*) was amplified using the primers flaBpro5XbaI and BblucPacI, and then cloned into pGEM-T Easy vector (Promega, WI) to form pGEM-*Bbluc*. After digestion with XbaI and PacI restriction enzymes, the PflaB-Bbluc fragment was ligated to the NheI and PacI digested pX*bbe02* to generate pX*bbe02Bbluc*. The streptomycin resistance cassette under *flgB* promoter (P*flgB-aadA*) was amplified using the primers 5FlgBpro and 3aadA. After restriction digestion with PacI of pX*bbe02Bbluc*, *aadA* were inserted to obtain pX*bbe02Bbluc-aadA*. The *bbe02* disrupting segments are flanked by at least 800 bp to facilitate double homologous recombination in the specific region of *B*. *burgdorferi*. The sequence of the plasmid confirmed the accuracy of our clone and was then used for transformation of the infectious, low passage N40 strain.

**Table 1:** PCR Primers and conditions used in this study.

Primer name	Sequence (5’ to 3’)	Expected PCR product size (bp)	PCR cycle parameters
BBE02Nde-long	CGC ATA TGA AAA CTA ATG ATA TCG TAA		95°C for 1 min
	AAA CAA ATA AT		50°C for 1 min
BBE02Xho-long	CGC TCG AGT TAT TTA TGA TAA AAA ATT	3834	72°C for 4 min
	TTA TTA TTT AGT AAA TAA TTA TC		
flaBpro5XbaI	TCT AGA CCG ATC GCC CTT CCC AAC AGT		95°C for 1 min
	TGC GCA GTG GAA GG		50°C for 1 min
BblucPacI	CGG TTA ATT AAT TAT TAT ACA GCA ATT TTA CCA CCT	1910	72°C for 2 min
5FlgBpro	GCT TAA TTA ACT TTT TTT TGA AGT GCC TGG		95°C for 45 sec
	CAG TAA GTT G		55°C for 45 sec
3aadA	GCT TAA TTA ATT ATT TGC CGA CTA CCT TGG	939	72°C for 1 min
	TGA TC		95°C for 30 sec
5BBE02mut	AAG ATA TCT AAA GAC AAG TAA TGT AAT	2225 or 3831	66°C for 30 sec
	AGA G		72°C for 4 min
3BBE02mut	CAT CGT CTT TAC TAT CTT TCA AAT CC		95°C for 30 sec
5BBQ67	AAT ATG TTT CAT TGT TTT ATA TCT TGG CTC	1407	60°C for 30 sec
3BBQ67	CCC TAT TGT TAG TTT TAT TGT TAG TAG TTT		72°C for 2 min

### Generation of *bbe02* mutants in N40D10/E9 strain of *B*. *burgdorferi*

*B*. *burgdorferi* strain N40D10/E9 (to be further referred as N40 in this paper) was transformed using pX*bbe02Bbluc-aadA* plasmid and colonies were selected on streptomycin (100 μg/ml) containing BSKII+RS plates. The mutants disrupted in *bbe02* gene are expected by double homologous recombination, such that the disrupted gene on the pX*bbe02Bbluc-aadA* plasmid replaces the wild-type *bbe02* located on lp25 plasmid. The clones obtained were designated as N40 *bbe02::Bbluc-aadA*. The selected clones were grown in 50ml BSKII+RS medium and genomic DNA isolated using previously described protocol [52]. PCR amplification using primers 5BBE02mut and 3BBE02mut, which flank the disruption site, was performed to confirm mutation of the *bbe02* gene and the absence of the wild-type *bbe02* gene in the selected *B*. *burgdorferi* mutant strains.

### Mouse model of infection

We have observed that both male and female C3H mice show similar Lyme disease manifestations when inoculated with our N40 strain [[46]; and data not shown]. Therefore, in this study we have only used female C3H mice. Since the median infectious doses (ID_50_) of our N40 has been shown to be <50 in our previous studies [46], we infected four-week old female C3H/HeJCr mice (National Cancer Institute) by subcutaneous inoculation of this strain and its *bbe02* mutant on the top of the right hind leg on the dorsal side at a dose of 10 to 10^3^ spirochetes per mouse for ID_50_ determination and 10^6^ per mouse for imaging when luciferase is present on the shuttle vector. Three (or four for inoculation dose of 10^3^) mice were used for each dose of infection. Mice were euthanized at four weeks of infection by CO_2_ asphyxiation and tissues were harvested for culture of the spirochetes and right joint and heart for histopathology. Skin at the injection site, ear, blood, urinary bladder, and left joint were transferred to tubes containing BSK-II+RS medium and antibiotic mixture for Borrelia (Sigma-Aldrich, St Louis, MO) and grown at 33°C. ID_50_ for N40 *bbe02::Bbluc-aadA* was determined by examination of live cultures recovered from the mouse tissues. For histological examination, heart and joint of each infected mouse was fixed in neutral buffered formalin, processed by routine histological methods, and scored blindly for arthritis severity, as previously described [7]. In repeat of the experiment, left joint was used for DNA isolation and quantitative PCR (qPCR) as previously described [53].

### Ethics Statement

Animal work at Rutgers University has approval from the Association for Assessment and Accreditation of Laboratory Animal Care (AAALAC). All mouse experiments were conducted under the protocol (11044D0514) approved by Rutgers-New Jersey Medical School Animal Care and Use Committee.

### *In vivo* imaging of bioluminescent *B*. *burgdorferi* in mice

In a pilot experiment, four-week old female mice were injected subcutaneously with approximately 10^6^ *B*. *burgdorferi* B31 strain harboring the shuttle vector pJSB175. Bioluminescent images were taken 10 minutes, 7 hours and 11 days after infection by IVIS (Perkin Elmer, Waltham, MA). At the start of infection, *B*. *burgdorferi* culture medium containing appropriate inoculum dose mixed with D-luciferin substrate was injected on the top of the right hind leg on the dorsal side and images captured within 10 minutes. For each subsequent imaging time point, 1.5 mg of D-luciferin dissolved in PBS was injected intraperitoneally as luciferase substrate per mouse and images were captured within 10 minutes after injection using IVIS 200 (Perkin Elmer, MA).

To investigate the possible effect of selective pressure exerted by streptomycin on retention of pJSB175 plasmid in *B*. *burgdorferi* during mouse infection, in the second experiment, six mice were injected subcutaneously with approximately 10^6^ *B*. *burgdorferi* strain B31 harboring the pJSB175. These mice were then divided into two groups of three mice each. One group was provided with drinking water supplemented with 5 mg/ml of streptomycin, and the other was provided with drinking water without antibiotics. Bioluminescent images were taken 10 minutes, 1 day, and 3 days after infection.

To determine ID_50_ of the strains, four-week old female mice were injected subcutaneously at a dose of 10, 10^2^, or 10^3^ *B*. *burgdorferi* strains without (for the wild-type N40) or mixed with (for N40 *bbe02::Bbluc-aadA* mutant) 1.5 mg of D-luciferin as recommended by the supplier (Caliper Life Sciences, Inc. Hopkinton, MA). The mice were also injected intraperitoneally with 1.5 mg of D-luciferin as luciferase substrate in 100 μl PBS for imaging at later time points. Bioluminescent images were taken with an exposure time of 30 seconds to 1 minute using IVIS-200 (Perkin Elmer, MA) on the day of inoculation, and at 7, 14 and 28 days post-infection. Captured images were further analyzed using Live Image Software (Perkin Elmer, MA). After four weeks of infection, mice were euthanized and tissues harvested as described above.

### Determination of transformation efficiency of *B*. *burgdorferi*

Transformation of N40 and N40 *bbe02::BBluc-aadA* cultures was conducted using 30 μg of shuttle vector pBSV2G by electroporation using standard protocol [54]. For selection, 80 μg/ml of gentamicin was added, and the culture was incubated at 33°C overnight again before plating in BSKII-RS medium supplemented with 80 μg/ml gentamicin. Frequency of transformation was calculated by counting the number of transformant colonies obtained on BSKII+RS plates per 5x10^8^ spirochetes.

### PCR and Southern hybridization to confirm the presence of *bbq67* gene

PCR primers 5BBQ67 and 3BBQ67 were designed to amplify an internal fragment from the *bbq67* gene of the B31 genome present on lp56 (Table 1). N40 strain lacks lp56 [27, 50]. This PCR was performed to test whether N40 genome possesses a homolog of *bbq67* elsewhere on the genome. Genomic DNA of B31 5A18 NP1 (genotype lp56^-^, lp28-4^-^, *bbe02*::Kan^r^) was included in this assay as a negative control, because this B31 derivative lacks the plasmid lp56 [32]. Genomic DNA isolated from B31 5A4, B31 5A18 NP1, and N40 cultures were digested with restriction enzyme EcoRI. DNA fragments were resolved by agarose gel electrophoresis, and transferred to Nytran SPC membrane (GE Healthcare Life Sciences, PA). Southern hybridization was carried out following the standard protocol to confirm the absence of *bbq67*-homolog in N40 genome. PCR product amplified from B31 5A4 genomic DNA by 5BBQ67 and 3BBQ67 primers was labeled with DIG using DIG High Prime DNA Labeling and Detection kit (Roche, IN). Hybridization and detection procedures were carried out according to manufacturer’s instructions.

## Results

### Plasmid vector containing the luciferase gene is unstable during infection in the absence of antibiotic selection

We first examined whether the luciferase gene located on a shuttle vector can be used for live imaging of *B*. *burgdorferi* spirochetes in Lyme disease susceptible C3H mice from inoculation state to persistent infection. Two clones that retained all endogenous circular and linear plasmids of *B*. *burgdorferi* strain, B31A3 were selected (data not shown). We did not observe any change in growth of these two clones and light emission increased with bacterial growth (data not shown). Surprisingly, bioluminescence appeared to increase at late stationary phase of growth after declining slightly at early logarithmic phase of growth (data not shown). Since luciferase expression is driven by the constitutively active *flaB* gene promoter, whose expression is not affected by growth conditions and media pH, using fresh and spent media at pH 6.5–6.8, provides optimum conditions for maximum luciferase activity. The firefly luciferase prefers pH ~6.6 for maximum activity [55]. Interestingly, spirochetes suspended in normal pH of *Borrelia* fresh medium (pH 7.6) showed least bioluminescence and the bacteria suspended in the fresh medium adjusted to pH 6.5 showed maximum light emission, indicating that pH 6.5 is optimal for *B*. *burgdorferi* codon-optimized luciferase (Fig 1). Relatively low levels of light emission by *B*. *burgdorferi* suspended in spent medium or culture in early stationary phase of growth at pH 6.8 are likely due to partial exhaustion of energy sources available in these media and decreased ATP levels in bacteria at this stage. ATP is essential for firefly luciferase activity. Thus, pH, energy source and ATP levels within spirochetes likely affect D-luciferin catabolism and light emission by *B*. *burgdorferi* codon-optimized firefly luciferase in BSKII+RS medium.

Although several studies have shown that disseminated infection by *B*. *burgdorferi* mostly occurs within 3–7 days after infection [21, 39, 56, 57], we were able to detect dissemination within 7h of injection (Fig 2A). However, bioluminescence in this mouse was no longer detectable from 24h to up to 11 days after inoculation (data not shown). In the second experiment, after subcutaneous injection of clone 3 that expressed firefly luciferase gene with a high dose of infection (~10^6^ spirochetes/mouse), infected mice were monitored daily by IVIS. Spirochetes in mice provided with antibiotic-free drinking water appeared to have lost the shuttle vector within a day and did not exhibit bioluminescence after 24h of infection, confirming results shown in Fig 2A. One mouse in this set died unexpectedly one day after injection. These mice did not show any bioluminescence 3 days after infection (Fig 2B, left panel). Three mice, provided with 5mg/ml of streptomycin in drinking water to ensure retention of the luciferase-containing shuttle vector, stopped drinking water and of these, two showed dehydration manifestations. They were euthanized two days after injection of *B*. *burgdorferi*. The remaining mouse, treated with streptomycin-containing drinking water, showed bioluminescence and disseminated infection by live imaging three days after injection (Fig 2B right). (All mice were euthanized three days after infection.) These results indicate that luciferase located on the *B*. *burgdorferi* shuttle vector is not an ideal choice for long-term live imaging of the infected mice.

### Confirmation of *bbe02* mutation in *B*. *burgdorferi* N40D10/E9 strain

**Fig 4:**
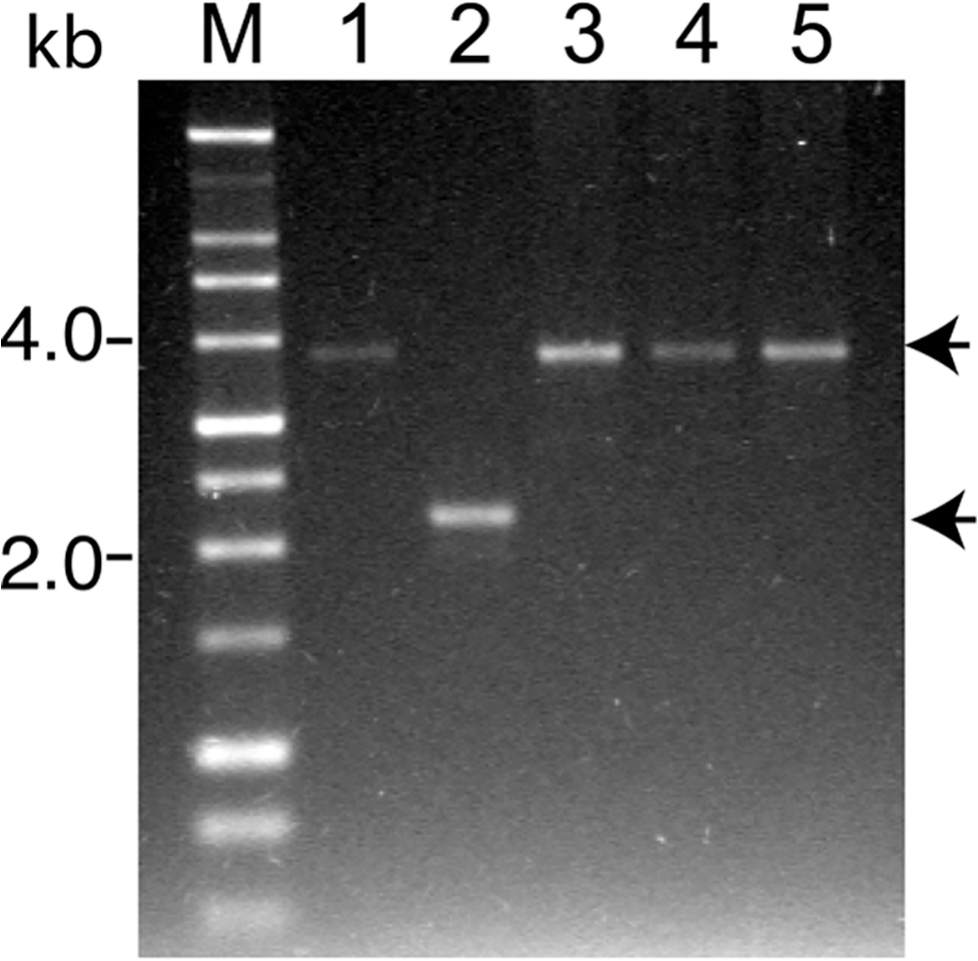
Confirmation of disruption of the *bbe02* gene in infectious N40 strains by PCR. Lane M is 1 kb size DNA ladder. For PCR, pX*bbe02Bbluc-aadA* plasmid was used as control template (Lane 1). PCR amplicons of 2225 bp was detected when genomic DNA of the wild-type N40 was used as template (Lane 2) while a single amplicon obtained from each of three N40 *bbe02::Bbluc-aadA* mutant clones of approximately 3831 bp indicates that disruption of *bbe02* gene occurred by double homologous recombination (Lanes 3 to 5).

To overcome the problem associated with shuttle vector encoded luciferase gene, we decided to disrupt *bbe02* with *Bbluc* and a streptomycin cassette (*aadA* gene) in tandem, to obtain stably bioluminescent *B*. *burgdorferi* by using streptomycin to select the mutants (Fig 3). We selected the N40 strain for stable expression of firefly luciferase because it shows much more pronounced inflammatory disease of heart and joints relative to the sequenced B31 strain. We can visually observe an inflammatory response and joint swelling depicting arthritic manifestations in mice infected with this N40 strain ([46, 58]; and unpublished work). After transformation of freshly prepared, competent N40 strain with pX*bbe02::Bbluc*-*aadA*, bacteria were plated in streptomycin- containing BSKII+RS medium (100μg/ml). Transformation efficiency was poor and four transformation attempts yielded only three clones that retained all N40 endogenous plasmids (data not shown). This is not surprising since transformation efficiency of N40 is lower than for infectious B31 strain ([34], personal communication from James Bono). Transformation of N40 D10/E9 strain to generate *bgp* mutants was first conducted by James Bono in Dr. Patricia Rosa’s laboratory and only three authentic mutants were obtained in that study [58]. Primers 5BBE02mut and 3BBE02mut were designed to amplify the region flanking the disrupted *bbe02* gene to differentiate between wild-type and mutated *bbe02* in Str^R^ clones obtained after transformation. Due to disruption of the *bbe02* gene with *Bbluc* and *aadA* (Str^R^ selection marker), the PCR amplicon in the *bbe02* mutant was expected to be larger (~3.8kb) than the wild-type N40 strain (2 kb). All three clones of N40 *bbe02::Bbluc*-*aadA* amplified a product of ~3.8kb size, indicating the presence of only the mutated *bbe02* gene (Fig 4) and confirming that disruption of the *bbe02* had occurred by double crossover. We randomly selected clone 3 for further experiments.

### Bioluminescent *B*. *burgdorferi bbe02* mutant strain has infectivity comparable to the wild-type N40 strain

Our previous studies have shown that ID_50_ of the wild-type N40D10/E9 is less than 50 [46]. To determine whether the infectivity of the *bbe02* mutant is comparable in the immunocompetent C3H mice, we injected a dose of 10, 100 and 1000 of each spirochete strain per mouse. The experiment was performed twice. Based on the culture recovery of spirochetes from different tissues that demonstrate colonization, the ID_50_ of bioluminescent N40 *bbe02::Bbluc-aadA* was 22 and 81 in the two experiments, similar to the parental N40 strain’s ID_50_ of 27 and 43 in two experiments (Table 2).

**Table 2:** Colonization of C3H mouse tissues by N40 D10/E9 or N40 *bbe02::Bbluc*-*aadA* strains examined four weeks after inoculation.

Strain	Inoculum	Recovery of *B*. *burgdorferi* from mouse tissues						ID_50_
		Blood	Right Ear	Injection site	Bladder	Joint	Total	
N40 D10/E9	10	0/3	1/3	0/3	1/3	NA	2/12	43
N40 D10/E9	10^2^	2/3	3/3	0/3	3/3	NA	8/12	
N40 BBE02::Bb*luc*-*aadA*	10	0/3	2/3	1/3	2/3	2/3	7/15	22
N40 BBE02::Bb*luc*-*aadA*	10^2^	0/3	3/3	1/3	3/3	3/3	10/15	
N40 BBE02::Bb*luc*-*aadA*	10	1/3	1/3	0/3	1/3	NA	3/12	
N40 BBE02::Bb*luc*-*aadA*	10^2^	1/3	2/3	0/3	2/3	NA	5/12	81
N40 BBE02::Bb*luc*-*aadA*	10^3^	0/4	4/4	3/4	3/4	NA	10/16	

### *In vivo* imaging of *B*. *burgdorferi bbe02* mutant displays spatial and temporal distribution to depict tissue colonization

**Fig 5:**
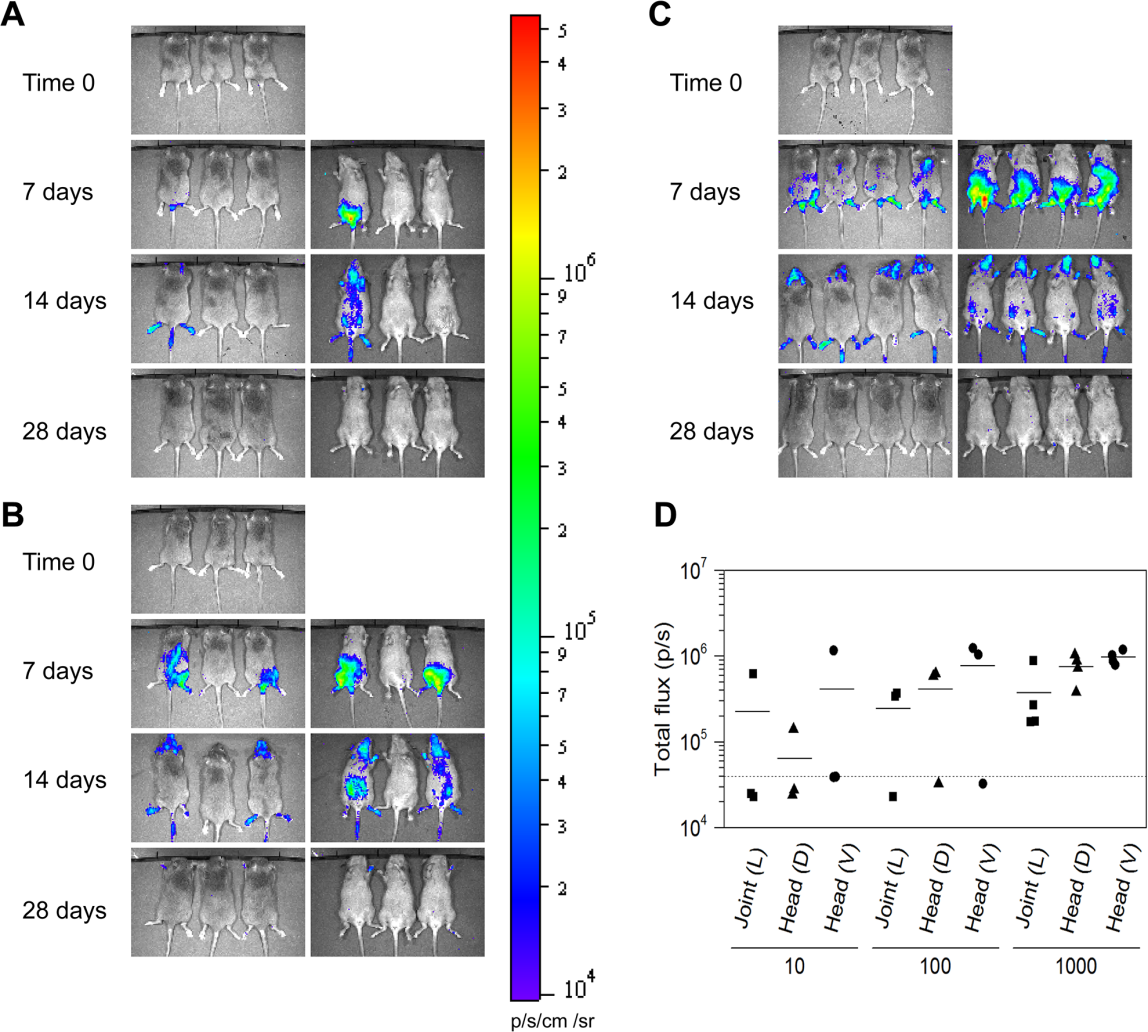
Spatial and temporal distribution of *B*. *burgdorferi* N40 *bbe02::Bbluc-aadA* in infected C3H mice. Real-time imaging of the same set of infected mice on the day of inoculation and also at 7, 14 and 28 days after infection shows the presence of live *B*. *burgdorferi*. For imaging, each mouse was injected with 100 μl PBS containing 1.5 mg of D-luciferin intraperitoneally. Images were taken with 30 seconds of luminescent exposure time. **(A)** Images of three C3H mice injected with 10 spirochete of N40 *bbe02::Bbluc-aadA* per mouse from dorsal (Time 0) or ventral (later time points) side are shown. Similar images are shown for mice infected with 10^2^ **(B),** or 10^3^ **(C)** N40 *bbe02::Bbluc-aadA* spirochetes. Absence of light emission after four weeks of infection indicates that adaptive immune response has reduced *B*. *burgdorferi* levels below the sensitivity of detection of IVIS 200. The same minimum and maximum luminescence range were used in all images as indicated by color spectrum scale. **(D)** Determination of the level of colonization in head and joint regions of C3H mice by bioluminescence quantitation at 14 days of infection. Background threshold is marked by a dotted line. Mice that remain uninfected showed luminescence signal below the threshold value after luciferase injections and thus, provide ideal controls for background signal.

Our *in vivo* results agree with elegant work of Hyde and coworkers [21] conducted previously in which a shuttle vector containing *bbe22* and codon-optimized luciferase gene was transformed into a B31 strain lacking the lp25 plasmid [21]. In both studies, live imaging of infected mice shows that dissemination of *B*. *burgdorferi* and colonization of various tissues occurs within a week. In fact, using IVIS we detected live *B*. *burgdorferi* colonizing various tissues particularly in the tibiotarsus seven days after infection, (Fig 5). Colonization of joints by optical imaging was not apparent in the study of Hyde and coworkers [21]. In our experiments, colonization of the tibiotarsus in the mice injected with an initial inoculum of 10 spirochetes was not observed after one week of infection (Fig 5A). After two weeks of inoculation, various tissues of the mice were heavily colonized, especially limbs, skin, brain, tail and lymph nodes (Fig 5). Colonization level appears to be at its peak at this stage of infection suggesting that spirochete clearance by adaptive immune response is not pronounced until this time point. These results agree with the finding of Hyde and coworkers [21]. However, four weeks after infection, live imaging detected minimal to no light emission suggesting clearance of spirochetes to levels close to or below the detection threshold of IVIS-200 for *B*. *burgdorferi* (Fig 5). We further quantitated luminescence in the infected tissues at different stages of infection. For simplicity of presentation, data for two doses of inoculation at the two-week time point are shown (Fig 5D). The background level of luminescence in control mice that did not get infected (at inoculation dose of 10 and 100) was <5x10^4^ photons per second per squared centimeter per steradian (p/s/cm2/sr) after substrate injection (Fig 5D, data not shown for mice infected with 10 spirochetes). Therefore, this photons value was considered the minimum threshold of detection of live spirochetes by optical imaging using IVIS-200. Luminescence quantification in the infected mice at 7 and 14 days post inoculation at each dose of infection showed values higher than the threshold of detection. Disseminated colonization of tissues was clearly discernible in the head and the left joint, i.e., distant sites since *B*. *burgdorferi* were injected on the right flank region. Light emission in identically marked areas in different mice showed that the number of live bacteria was significantly higher in the head region at day 14 as compared to day 7, post inoculation (p<0.05 for dorsal side and p<0.005 for ventral side as determined by t-test). However, colonization levels did not change significantly between days 7 and 14 in left joints (p = 0.164), which also depict a disseminated distant site. Luminescence measurements indicated that at four weeks of infection, spirochete numbers were below the threshold of detection (data not shown). Supporting this premise is our ability to recover bioluminescent *B*. *burgdorferi* from the urinary bladder, joint, and ear by culture at this time point, indicating the presence of live spirochetes in different tissues (Table 2). Furthermore, qPCR at four weeks of infection showed low spirochete burden in the ear and the distant, left joint (Table 3). Previously, Dr. Skare’s laboratory showed a high correlation between detection of spirochete burden by bioluminescence and qPCR [21]. Thus, even though spirochetes are present in various tissues, the numbers of *B*. *burgdorferi* colonizing these tissues are below the sensitivity of detection in C3H mice of the currently available IVIS machine. Disseminated infection was also comparable between the two strains (Table 2, Fig 5, and [46]).

**Table 3:** Real-time PCR quantitation of *B*. *burgdorferi* genomic copy number at four weeks of infection in the tissues of mice inoculated with N40 *bbe02::Bbluc-aadA*.

Inoculum	Mouse	Copies of *B*. *burgdorferi* genomic DNA per 200 ng of mouse DNA	
		Ear	Joint
10	I	ND	139.74
10	II	ND	ND
10	III	ND	ND
10^2^	I	ND	149.24
10^2^	II	ND	ND
10^2^	III	137.55	ND
10^3^	I	ND	94.39
10^3^	II	110.45	60.06
10^3^	III	47.55	52.72
10^3^	IV	98.63	26.81

Data normalized to obtain *B*. *burgdorferi* copy number in 200 ng of DNA (10^5^ copies of mouse nidogen gene) in respective tissues. ‘ND’ DNA not detected, i.e., below the detection limit.

### Comparative analysis of pathogenesis of N40 strain and its *bbe02* mutant

**Fig 6:**
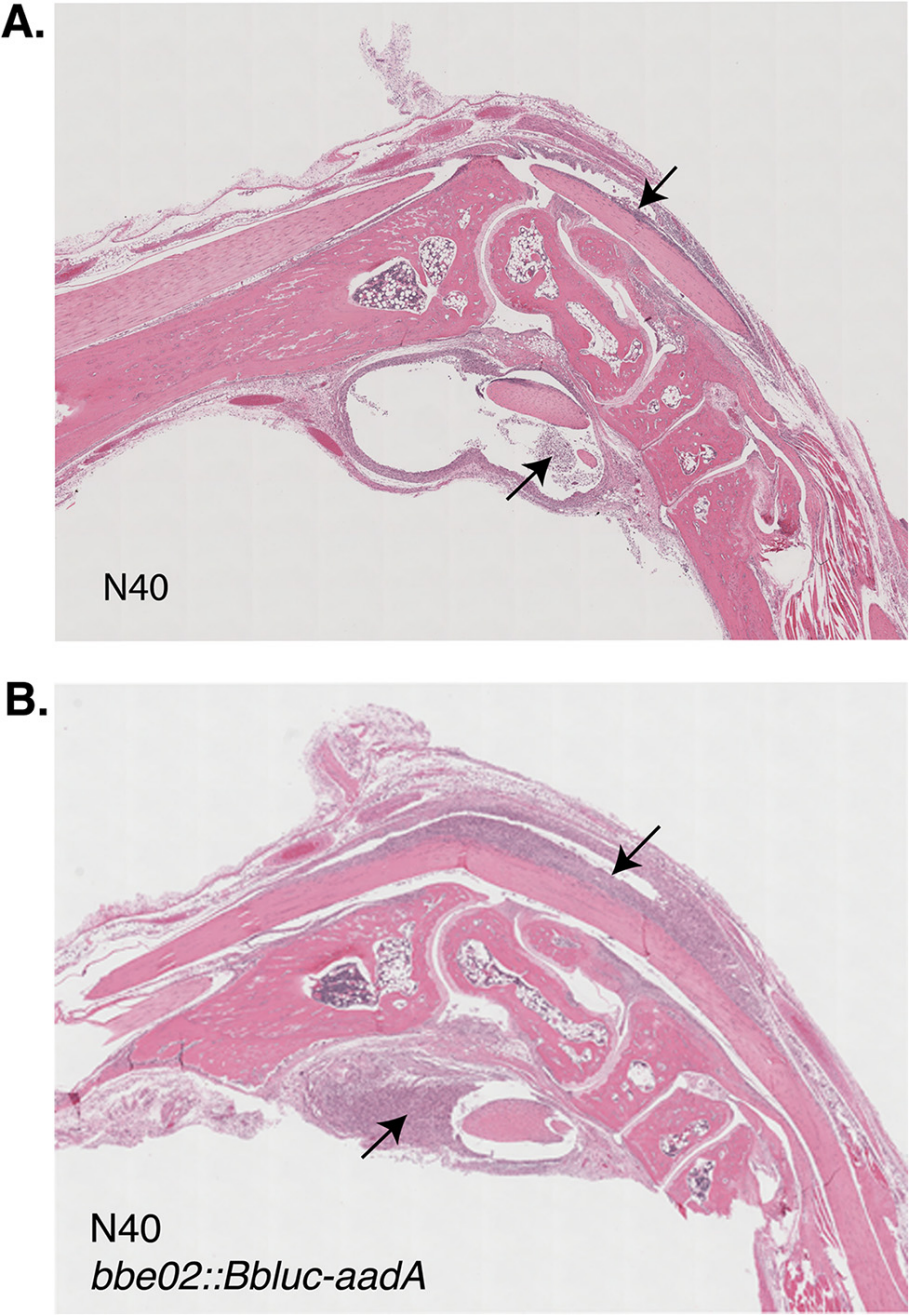
Histopathological examination of tibiotarsal joint of the infected C3H mice. Significant joint swelling is present up to four weeks of infection, even though colonizing bacterial number is reduced. These observations are consistent with histopathological examination of joints infected with both the wild-type N40 (A) and its *bbe02* mutant (B) and show significant inflammation of flexor and extensor tendon sheaths (marked by arrows).

Colonization of heart and joints of both humans and C3H mice with infectious *B*. *burgdorferi* results in carditis and arthritis, respectively [3, 7]. We were able to discern arthritic manifestations on visual observation of infected mice by both strains (infected mice in Fig 5). To determine the actual inflammatory response to *B*. *burgdorferi* wild-type and mutant strains, we further examined the heart and joint sections stained with hematoxylin-eosin stain. One representative sample with a pronounced inflammatory response in the tibiotarsal joint of mice infected with each *B*. *burgdorferi* strain is shown (Fig 6). Histological examination of the tibiotarsal joint and the heart did not reveal a significant difference in the severity of inflammation caused by bioluminescent N40 *bbe02::Bbluc-aadA* and the wild-type N40 strains four weeks after infection (Fig 6, and data not shown).

To further determine the association of colonization with Lyme disease manifestations, coded slides with sections of hearts and joints of infected mice were scored for inflammation in a blinded manner. Results of the histopathological evaluation are shown in the Table 4. Some mouse-to-mouse variation at each infection dose was observed as expected; and inflammation of the heart and knee were noticed in all doses of infection in both wild-type N40 strain and its *bbe02* mutant. Furthermore, moderate to severe inflammation of the tibiotarsal joint was observed in mice infected with both strains of *B*. *burgdorferi*. Thus, despite relatively low numbers of spirochetes present in joints, inflammation is quite pronounced at four weeks of infection (Tables 3 and 4).

**Table 4:** Histological examination of hearts, right knee and tibiotarsal joints of C3H mice infected with N40 D10/E9 or N40 *bbe02::Bbluc-aadA*.

Strain	Inoculum	Mouse	Heart	Right knee	Right tibiotarsal
			inflammation	inflammation	joint inflammation
N40 D10/E9	10	I	+	-	++
	10	II	-	-	-
	10	III	-	-	-
	10^2^	I	-	+	+++
	10^2^	II	-	+	++
	10^2^	III	+	+	++
N40 BBE02::Bb*luc-aadA*	10	I	+	+	+++
	10	II	-	-	-
	10	III	-	-	-
	10^2^	I	-	-	++
	10^2^	II	+	+	+++
	10^2^	III	-	-	-
	10^3^	I	+	+	+++
	10^3^	II	+	+	++
	10^3^	III	+	-	++
	10^3^	IV	+	+	++

For each mouse infected with specified inoculation dose, heart or knee inflammation was recorded as either “-”(no inflammation) or “+” (inflammation). Inflammation of tibiotarsal joint was scored from “-” (no inflammation), “+” (mild inflammation), “++” (moderate inflammation) to “+++” (severe inflammation).

### *B*. *burgdorferi* N40 strain lacks the second restriction modification system homologous to *bbq67* located on lp56 of B31 strain

**Fig 7:**
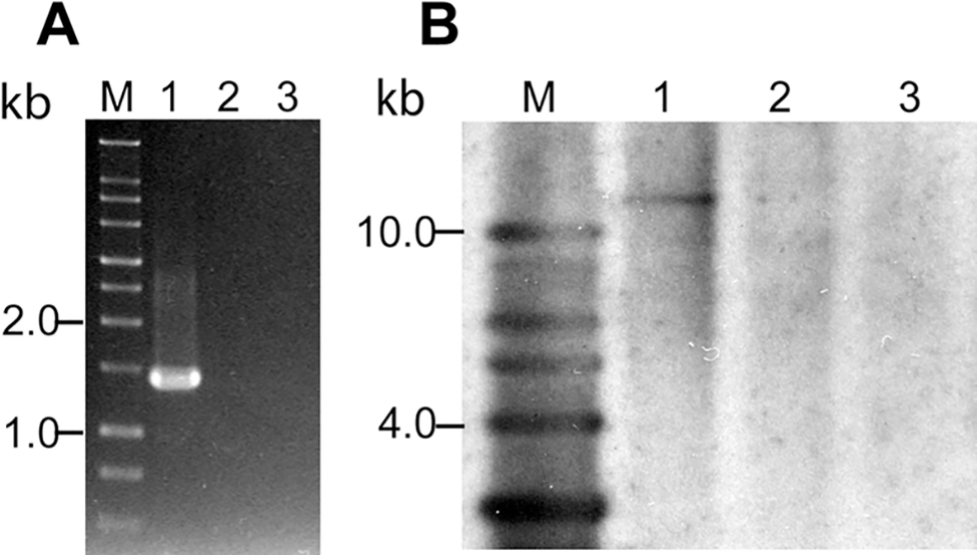
PCR and Southern hybridization confirm the absence of the *bbq67* gene in the N40 strain. **(A)** Lane M is 1 kb size DNA ladder. PCR amplification using the genomic DNA of B31 5A4, B31 5A18 NP1, and N40 D10/E9 strains as templates in lanes 1 to 3, respectively shows that *bbq67* is present only in B31 5A4 strain. B31 5A18 NP1 strain lacks lp56 and hence, also *bbq67*. **(B)** Lane M is 1 kb DNA ladder. Southern hybridization of genomic DNA of B31 5A4 (lane 1), B31 5A18 NP1 (lane 2), and N40 D10/E9 (lane 3) digested with EcoRI using the DIG-labeled *bbq67* PCR product from B31 5A4 strain PCR confirmed that N40 and B31 5A18 NP1 lack *bbq67* gene.

We previously showed that lp56 is absent in our N40 D10/E9 strain [50]. Sequencing of the N40 strain also showed that lp56 is missing in this strain [27]. Therefore, we expected that *bbq67*, which is located on lp56 of the B31 strain, could also be absent in our N40 strain. Interestingly, a smaller *bbe29* gene (1.8 kb in size) located on lp25 of B31 shows significant sequence homology (73% identity) to *bbq67* gene. To determine if N40 indeed lacks gene equivalent to complete *bbq67* gene, we designed primers in the region of *bbq67* that is different from *bbe29*. A PCR amplicon was detected only in B31 5A4 indicating the presence of *bbq67* in this strain and not its derivative B31 5A18 that lacks lp56. In addition, no amplicon was obtained from N40 strain genomic DNA (Fig 7A). To eliminate the possibility that a *bbq67* homolog is present in the N40 strain but shows sequence variation from the *bbq67* of B31 strain, we conducted Southern hybridization. Southern hybridization of blot prepared from total genomic DNA digested with EcoR1 using a probe prepared using a PCR amplified segment from B31 5A4 strain confirmed that N40 indeed lacks a *bbq67* homolog (Fig 7B).

### Transformation efficiency of a N40 mutant with disrupted *bbe02* is significantly higher

A major reason for inserting *Bbluc* for *bbe02* disruption was to make a stably bioluminescent strain and overcome the inhibitory effect of the *bbe02*-encoded restriction modification system on transformation of *B*. *burgdorferi*. Transformation efficiency of N40 is even lower than the B31 strain. Indeed, only our group, originally with the help of Dr. Patricia Rosa’s laboratory at Rocky Mountain Laboratory [58], has been successful in obtaining transformants in this strain. In both of our experiments, transformation of the N40 *bbe02::Bbluc-aadA* strain followed by plating on BSKII+RS agar plates resulted in colonies (Table 5). Lack of transformants in N40 in these experiments was not surprising since it took us four attempts to get *bbe02* mutants in this strain. Thus, the N40 *bbe02::Bbluc-aadA* strain has significantly higher transformation efficiency. Ability to obtain transformants in highly infectious N40 genetic background is very important since we, and others are more likely to obtain a specific mutant in the first attempt in the future when this *bbe02* mutant is used as a parental strain.

**Table 5:** Transformation efficiency increased in the N40 *bbe02::Bbluc-aadA* strain, compared to the wild-type strain N40 D10/E9.

Strain	Number of gentamicin-resistant colonies or wells per 5x10^8^ spirochetes	
	Experiment 1	Experiment 1
N40 D10/E9	0	0
N40 *bbe02::Bbluc-aadA*	10	32

Plasmid pBSV2G DNA is used to transform *B*. *burgdorferi* strains by electroporation. Transformation frequency is calculated by the number of gentamicin-resistant colonies per 5x10^8^ spirochetes, from a total of two experiments.

## Discussion

Previous studies have either addressed the issue of inhibition of transformation and uptake of foreign DNA due to two restriction modification systems of *B*. *burgdorferi* or live imaging to study the role of a particular spirochete protein, such as DbpA, during infection of mice [21, 31, 33, 34, 38, 59]. This is the first study to combine both approaches to generate a *B*. *burgdorferi* infectious strain stably expresses luciferase suitable for *in vitro* and *in vivo* live imaging of the spirochetes and also shows higher transformation efficiency.

In our studies, expression of codon-optimized firefly luciferase in B31 strain through gene inserted into a shuttle vector addressed this issue. However, the plasmid was unstable during infection in the absence of antibiotic treatment, as detected by a loss of light signal in live imaging of infected mice within 24h, and by PCR (data not shown). The loss of the shuttle vector during mice infection has been previously described [60]. On repeating this experiment again using a high inoculum, 10^6^ spirochetes/mouse, we compared infected mice with and without treatment with streptomycin three days after infection. However, mice provided with water containing streptomycin did not seem to like the taste and stopped drinking water. Therefore, our study could not continue beyond three days of infection. We have since learned that inclusion of artificial sugar such as commercially available ‘Splenda’ or ‘Equal’ in drinking water along with the antibiotics can overcome this problem. Therefore, plasmid-borne luciferase gene can possibly be used under these conditions. However, the major problem associated with the continuous use of antibiotics still remains. Several studies indicate that antibiotic treatment of mice for a long time can severely affect the native microbiome of the host leading to unintended consequences [61–64]. Therefore, maintenance of luciferase expressing gene located on the shuttle vector by continuously providing antibiotic is less than ideal for long-term *B*. *burgdorferi* infection experiments in mice.

Genetic manipulation of *B*. *burgdorferi* remains difficult due to low transformation efficiency of the infectious strains [25, 26]. Previous work identified two Type II restriction-modification systems, encoded by the genes *bbe02* and *bbq67*, as the features responsible for low transformation efficiency of the sequenced B31 strain [32–35]. Loss of these two genes did not affect infectivity of the Lyme spirochetes. We decided to generate stably bioluminescent infectious *B*. *burgdorferi* strain in this study that also exhibits high transformation efficiency. We selected N40 D10/E9 strain to accomplish this goal because we can visually observe development of arthritic manifestations caused by this strain in C3H mice [46]. Furthermore, we have previously shown that this strain lacks lp56 on which *bbq67* is located in the B31 strain [50]. We obtained the mutant by disrupting *bbe02* with *Bbluc* and streptomycin resistance cassette in tandem. The mutant displayed bioluminescence that showed association with spirochetal burden in infected tissues. Furthermore, due to stability and essential role of the lp25 plasmid in *B*. *burgdorferi*, luciferase was always retained during infection as indicated by recovery of spirochetes from mice even in the absence of antibiotic selection (data not shown). Our studies with the N40 strain agree with previous findings in the B31 strain where the loss of *bbe02* does not affect infectivity of *B*. *burgdorferi* [32–34, 47]. Infectivity of the wild-type N40 was not significantly different from that of the mutant as indicated by ID_50_ of these two strains. In addition, we were able to observe equivalent levels of spirochete dissemination and disease manifestations in C3H mice, by the wild-type and *bbe02* mutant strain. These results confirm that even in N40, *bbe02* disruption retains full infectivity and Lyme pathogenesis capability similar to the B31 strain that contains mutation in *bbe02*. Although some researchers prefer to use BALB/c mice for their experiments with bioluminescent *B*. *burgdorferi* [21], we found that young C3H mice (3–4 weeks old) for infection allows detection of light emission by live bacteria in the infected tissues, and exhibit pronounced disease manifestation, as reported in early studies on Lyme disease using mouse models of infection [8]. Furthermore, despite the agouti fur of C3H mice, we were able to detect luminescence representing disseminated infection in our experiments.

Different researchers have either used B31 strain derivatives lacking lp25 or lp56 for generation of mutants or specific mutants defective in *bbe02* and *bbq67* as parental strains to assess transformation efficiency and infectivity associated with these plasmids [31, 33, 34, 38, 59]. Since N40 strain lacks lp56 [50], we hypothesized that *bbq67* is absent in this strain. By confirming the absence of *bbq67* in N40 strain in this study by PCR and Southern hybridization, we predicted that our bioluminescent strain with *bbe02* mutation would display higher transformation efficiency. Although we cannot rule out the presence of another novel restriction modification system in this N40 strain, we observed a significant increase in transformation efficiency in our bioluminescent strain indicating only a minor role played by other, yet unknown restriction modification system, if any present in this strain.

Successful generation of a bioluminescent, highly infectious *B*. *burgdorferi* with increased transformation efficiency in this study offers an ideal parental strain to study Lyme pathogenesis in mouse model of infection. Although *B*. *burgdorferi* genes located on various plasmids are often important for either tick colonization, or for Lyme pathogenesis and immune-evasion in the mammalian hosts, their biological functions could not be determined due to their rather unique sequences [37]. For this reason, we did not want to generate an N40 strain derivative that lacks lp25 to increase transformation efficiency in our studies. To fully understand the molecular basis of Lyme pathogenesis, it is important that studies are carried out with the strains other than B31. Therefore, we have generated an N40 strain that retains its endogenous plasmids and maintains its high pathogenicity. By using our more competent, isogenic bioluminescent N40 strain for site-directed or random transposon mutagenesis, future functional assessments of unique *B*. *burgdorferi* proteins during infection will be much improved. Since the signature-tagged, Himar1 transposon of *B*. *burgdorferi* uses gentamicin cassette for clonal selection [43], we have specifically used a streptomycin cassette for selection of our *bbe02* mutant. Therefore, this mutant can immediately serve as a model strain for generation of defined transposon mutants for investigation of genes required for *B*. *burgdorferi* pathogenesis.

## Conclusions

Although restriction modification system affecting transformation capability of *B*. *burgdorferi* has been reported previously [31–34], this study describes for the first time, the generation of a highly infectious Lyme spirochete strain that shows higher transformation efficiency and also can be examined by In Vivo Imaging System in the live infected mice to determine tissue colonization. This *B*. *burgdorferi* strain can be used as a parental strain for generation of the site-specific mutants by various researchers in the future.
